# Experiments on the Electro-Galvanic Process for Depositing Plates for Artificial Teeth

**Published:** 1851-10

**Authors:** A. Hill


### ARTICLE III.

*Experiments on the Electro-Galvanic Process for Depositing Plates for Artificial Teeth. Read before the American Society of Dental Surgeons, at their Twelfth Annual Meeting, held in Philadelphia, Aug.* 5, 6 *and* 7, 1851. By A. Hill, D. D. S.

Gentlemen of the American Society:

Agreeably with the request which you were pleased to make, at the last annual meeting of this society, I herewith submit to you the results of some of my experiments in electro-plating; or rather, in depositing plates for mounting artificial teeth by the electro-type process.

Some eight or nine years ago, I conceived the possibility of depositing metallic plates, upon plaster or metallic models of the mouth for dental purposes. But being ignorant of the process, I did not attempt a practical solution of the matter at that time. Shortly subsequent, however, I called on some scientific gentlemen, in the city of New York, who I supposed to be well acquainted with the electro-type process, and on inquiry satisfied myself that the thing was not sufficiently developed to warrant a trial. And as from the best sources of information within my reach, there appeared to be no probability of success, the matter was deferred until an opportunity might occur for experiment.

About two years since, I became acquainted with a gentleman who was practically engaged in the electro-plating business, and it occurred to me, that here was a chance for experiment. I accordingly laid the whole matter before him, and obtained a favorable opinion as to the success of the undertaking. We immediately commenced our experiments, the results of which I now proceed to detail.

The first thing in order was to prepare my models. This was done in the ordinary way, with calcined plaster of paris, with a small copper wire to serve as a connection with the battery, running diagonally through the model, with the end slightly projecting in the palatine arch. This done, the next thing was to render the plaster model a non-absorbent.

For this purpose, after trying various experiments, I found that a solution of gun cotton in sulphuric ether, answered the best purpose. Copal, and other varnishes, although answering a tolerable purpose, we found would not stand the strong action of the alkaline solutions when immersed in them. But the gun cotton solution, commonly known as "liquid cuticle" was sufficient for this purpose.

After this was spread entirely over the model, with a brush, and suffered to become perfectly dry, the next thing was to make a conducting surface. Here, the greatest difficulty presented itself. Plumbago, which is ordinarily used, or rather recommended, for such purposes, we found would not answer. Various experiments were resorted to, to accomplish this object. The following, however, was most successful. After drawing a line with a pencil, around such parts of the model as we wished to have covered with the plate, we took a small camel's hair pencil brush, and laid over a thin coating of sizing, then upon the sizing, we would lay on gold or silver foil. This process requires much care, inasmuch as any imperfection or blemish is certain to be copied in the deposition, or plate. This was allowed to dry, after which, it was ready for the battery.

Instead of the foil thus applied, we sometimes used bronze, which answered a good purpose, especially where copper is to be deposited. Much care and practical experience is requisite to make a firm, solid and tenacious plate. The power of the battery must be delicately graduated, or the deposit will be rough and coarsely granulated. A gentle movement of the solution is also requisite, in order that the deposit may be uniform and even. After the plate had acquired sufficient strength and thickness, it was removed from the model, thoroughly cleansed, and then immersed, so as to receive a deposition on both sides. In this way a sufficient thickness may soon be obtained. Frequent removal and scouring is necessary to prevent an irregular granulation.

In this way, I have succeeded in forming several plates, of sufficient thickness and strength to be worn in the mouth; some of which I had the pleasure to exhibit before this society, at its last annual meeting. For the sake of economy, my experiments were made with silver and copper. I found that copper could be deposited with the greatest facility of either of the metals, upon plaster. I, therefore, conceived the idea, that copper might be used as a basis for the other metals, affording strength while it would likewise lessen the expense. I, therefore, took an impression of a lady's mouth, for an entire set of teeth. Made a deposit of copper, as above described, then cleaned the plate, and deposited a coat of pure silver over its entire surface; upon this, I mounted the teeth, using soft solder to secure them to the plate, after which they were again immersed in the battery, and received another coating of pure silver.

The fit was most perfect, and my patient delighted. By the way, I should mention, that this lady was the wife of the gentleman with whom I was experimenting, and of course knew the nature of our experiments. Here, then, I had high hopes of success. But a circumstance developed itself which soon taught us another lesson. In wearing this plate, a galvanic action took place, which seemed fatal to this particular feature of our enterprise.

But, I have become well satisfied by much reflection, that if this process is conducted in a perfect manner, no galvanic action can result. And for this very good and sufficient reason, viz. that where the copper is perfectly covered and protected from fluids by the silver, no such action can take place, as it is essential that the fluids of the mouth act upon both metals, in order to produce it.

This plate, mounted with teeth—the first in all probability ever worn—has now been used more than a year, although fresh deposits of silver have been from time to time made upon it, as I understand.

The use of copper (if it can be used in this way) will overcome, and obviate several difficulties.

1st. It can be deposited with greater facility, and therefore shortens the time of the process.

2nd. It diminishes the expense, and

3dly. It can be annealed without danger of warping, or blistering. And finally, if you add a coat of pure gold to the whole piece, I cannot imagine any harm that can come of it.

During the past year, circumstances have transpired which rendered it extremely inconvenient for me to pursue these experiments. And I regret exceedingly, that I am obliged to report so little progress in this very interesting department.

That plates can be deposited in this way, ensuring a more perfect adaptation to the mouth, while at the same time it relieves the profession of the entire process of swaging, I have already demonstrated. That it can be done with sufficient facility and economy to supersede the old method, remains to be seen. I cannot but regard the experiment as a fine field for instructive and useful study, and worthy the attention of every member of the dental profession.

I am of opinion, that models made of D'Aartes' mineral, or fusible metal, may be used instead of plaster, and thus obviate the difficulties of forming a connecting surface with that material.

When models of this metal are used, and a sufficient body of the plate deposited, the model, or casting, may be melted away, leaving the plate unharmed, annealing it at the same time.

I cannot doubt that the time will come, and that too, before long, when, in our large cities, the dentists will have nothing to do towards preparing a plate, but to make his casting, and draw around it the line for the borders of his plates, and leave it with the electro-platers.

It would be entirely superfluous and unnecessary for me here to enter upon a labored description of the ultimate advantages of this process, if once rendered practicable. They are so obvious as to suggest themselves to every reflecting member of our profession; and so great as to inspire fresh hopes of advantage in every case.

The metals deposited in this way, must necessarily be *pure*. An entire gold plate, therefore, could not be used to advantage, as pure gold is too soft. But pure gold may be deposited upon pure silver, of sufficient thickness to answer every purpose, and at the same time, be less objectionable than the ordinary rolled plate, now in use, as to any galvanic, or corrosive action in the mouth. It should be remembered, that all metals deposited by this process, require annealing, as they come from the battery hard and exceeding brittle.

In conclusion, gentlemen, allow me to express the hope, that this subject may be taken up by some member of the profession more competent than myself, and prosecuted to a successful termination; and if I can impart any hint or item of information that will contribute to this result, it will give me pleasure so to do.

